# No State Licence Given to Murder

**Published:** 1917-08-18

**Authors:** 


### THE MASSACRE OF THE INNOCENTS.

### No State Licence Given to Murder.

A discussion has appeared in the *Daily Mirror* concerning the right, duty, or privilege of a doctor to put to death or allow to die a child that is born deformed or imperfect. The following statement is put by the *Daily Mirror* into the mouth of an anonymous medical man, and may, perhaps, have been made by one. Similar sentiments are often expressed both by medical and non-medical persons:

### A Libel or an Outrage?

**It is, of course, an extremely delicate subject, and it needs plain speaking to justify the doctrine that the unfit should not be allowed to survive. There are children whose mental and physical disabilities are so terrible that it would have been an act of mercy to have let them die at birth. The great objection is that it might lead to the criminal destruction of healthy children under the mistaken assumption that they are unfit. It is recognised that a doctor or a nurse can and does save the life of an unfit child who might die but for the measures taken to assist [? save] life. But knowing that the child must be a useless member of society and will suffer terribly throughout life, neither doctor nor nurse can seriously justify its existence. Personally, and it is the view of the profession generally, I think such lives (where the evidence of the unfitness is clear) should not be saved in the interests of both the community and the individual.**

Another, or perhaps the same, anonymous or imaginary medical authority is quoted as saying:

**It is merely cowardice for the future of our professional career that prevents us doing what this American surgeon has done. I think a change will take place, for it has been established on the battlefield that the giving of death is more merciful in many cases than the giving of life, and this has been acted up to.**

### Some "Horrible Perversions of the Language."

In former times, when more attention was paid to the general and preliminary education of youths who were to enter the medical profession, we could have pointed to the atrocious travesty of the English language in which these sentiments are expressed as proof positive that they were not uttered by any member of the medical or either of the other learned professions. We admit with sorrow that this inference can no longer be drawn. Such horrible perversions of the language cannot now be regarded with certainty as mere journalese, and may have been uttered by qualified medical men, and even by University graduates. But passing the form in which they are placed before us, the sentiments themselves demand consideration, since there is a good deal of loose thought of the kind floating about, and one often hears, from medical and other persons, opinions of the expediency of medical practitioners putting to death, for one reason or another, patients of this class or that. It may be well, therefore, to straighten the matter out, and clear it up.

### A Practitioner's Limitations.

Medical practitioners are entrusted by the State with licence to practise physic—that is to say, to practise the art of the physician for the alleviation of pain and for the prolongation of life. They are not entrusted or authorised by the terms of their licence to use for any other purpose the knowledge of medicine that they acquire with the sanction, and to some extent with the assistance, of the State. They are licensed to heal; they are not licensed to murder. Let there be no mistake. Let the matter be put in plain terms, and it will be seen that what is proposed by the utterances we have quoted is that medical practitioners shall use their medical knowledge to commit murder, or shall in their discretion refrain from applying their medical knowledge, so that murder, or, at the least, manslaughter, shall result. By virtue of the knowledge that he has acquired, the State grants to the medical practitioner a licence which places him in a fiduciary position towards his patients, and leaves him no discretion as to the function he is to discharge. He is to save life if he can; he is to prolong life to the utmost of his power; and to take upon himself a discretion as to whether he shall exercise this function or reverse it is a gross and abominable dereliction of duty. The State has given him no licence to murder.

### Citizens' Lives and State Rights.

The State, as representing the community as a whole, has a complete right to demand the life of any of its citizens if that life is detrimental to the community, or if the paramount interest of the community requires the sacrifice of that life. This is the principle that underlies the right of the State to execute for murder or treason, and to send its citizens to battle. But a life may not be taken or forfeited without the express sanction of the State, uttered by its responsible official entrusted by the State with the duty of acting in such cases. To sanction, or even to wink at, private murder would be utterly destructive of the community that tolerated such a practice. It would be a return to barbarism, and would ensure the speedy disintegration of the community. If deformed and defective children are to be executed, they must be executed not by those who are entrusted with privileges in order that they may prolong life, but by the solemn act of the State after investigation and decision by proper officers appointed by the State for the purpose.

### A Libel on America?

We cannot credit that United States law would suffer a medical practitioner in America to destroy child or other life on the ground of its unfitness. Nor do we credit the statement that the American profession would tolerate qualified practitioners publicly indulging in organised murder, under the cloak of clearing the cities of all unfit children or adults, when discovered as members of any community. The trend of the best opinion in America, as we have known it, is all the other way. What is Sir William Osler's opinion and view?

